# CT‐augmented digital tomosynthesis image reconstruction in image‐guided bronchoscopy interventions

**DOI:** 10.1002/mp.17551

**Published:** 2024-12-03

**Authors:** Fatima Saad, Robert Frysch, Sylvia Saalfeld, Stephan Kellnberger, Jessica Schulz, Rebecca Fahrig, Krish Bhadra, Andreas Nürnberger, Georg Rose

**Affiliations:** ^1^ Institute for Medical Engineering Otto‐von‐Guericke University Magdeburg Germany; ^2^ Research Campus STIMULATE Otto‐von‐Guericke University Magdeburg Germany; ^3^ Institute for Medical Informatics and Statistics University Hospital Schleswig‐Holstein Kiel Germany; ^4^ Advanced Therapies, Innovation Siemens Healthcare AG Forchheim Germany; ^5^ CHI Memorial Rees Skillern Cancer Institute Chattanooga Tennessee USA; ^6^ Data and Knowledge Engineering Group, Faculty of Computer Science Otto‐von‐Guericke University Magdeburg Germany

**Keywords:** digital tomosynthesis, computed tomography, interventional bronchoscopy, transbronchial needle biopsy, image reconstruction, prior‐aided reconstruction

## Abstract

**Background:**

Transbronchial needle biopsy is crucial for diagnosing lung cancer, yet its efficacy depends on accurately localizing the target lesion and biopsy needle. Digital tomosynthesis (DTS) is considered a promising imaging modality for guiding bronchoscopy procedures due to its low radiation dose and small footprint relative to cone‐beam computed tomography (CBCT). However, the image quality of DTS is still not sufficient for an accurate guidance, mainly due to its limited‐angle acquisition.

**Purpose:**

Preoperative computed tomography (CT) scans are often performed prior to bronchoscopy interventions for diagnosis or to plan the procedure. The CT images are of high quality and are characterized by a high spatial resolution compared to intraoperative DTS images. These patient‐specific prior CT images and the intraoperative DTS images share a fair amount of anatomical information. The main differences only stem from patient positioning and respiratory motion. When these differences are addressed properly, prior CT images augment intraoperative DTS image reconstruction with strong prior knowledge and potentially enhance DTS image quality.

**Methods:**

We propose in this work a prior‐aided DTS image reconstruction technique leveraging prior CT images to improve DTS image quality. This technique is based on a recently published deformable CT‐to‐DTS image registration algorithm which is customized for bronchoscopy interventions. The main idea is to register a prior CT image to an intermediate DTS image reconstructed using the standard iterative algebraic reconstruction technique (ART), then to re‐reconstruct the DTS image using ART and the registered prior CT image as a first estimation.

**Results:**

The proposed prior‐aided reconstruction method was tested on a physical phantom and six patient bronchoscopy datasets. Real DTS data acquired with a pseudo‐linear (PL) scan geometry and simulated DTS data generated according to a spherical ellipse (SE) scan geometry were considered. Results evaluated qualitatively by visual inspection and quantitatively by computing Pearson's correlation (PC) with respect to the reference CBCT images suggest significant improvements in image quality using the prior‐aided DTS reconstruction compared to the standard zero‐initialized ART reconstruction. PC coefficients of the six patient datasets were on average 0.64±0.13 and 0.55±0.13 using a zero‐initialized ART reconstruction with SE data and PL data, respectively, and 0.82±0.09 and 0.72±0.09 using the proposed prior‐aided reconstruction with SE data and PL data, respectively.

**Conclusions:**

While the initial estimation in iterative reconstruction algorithms is often overlooked, we proved that initial estimation is of critical importance in DTS image reconstruction and we have demonstrated the profound advantages of integrating prior CT images in intraoperative DTS image reconstruction. CT‐augmented DTS offers a viable alternative to CBCT in guiding bronchoscopy interventions at a fraction of the radiation dose. Further clinical studies are needed to validate improved diagnostic yield.

## 1 INTRODUCTION

Lung cancer is the leading cause of cancer deaths globally; in fact, lung cancer causes more deaths than breast, prostate, and colon cancers combined.^1^ Bronchoscopic image guided transbronchial biopsies play a major role in the diagnosis of lung cancer. However, the diagnostic yield of these procedures depends on precisely identifying the location of the target lesion and the biopsy needle during the intervention. Posing lower radiation dose to the patients and imposing smaller scan footprints compared to cone‐beam computed tomography (CBCT) on the one hand, and providing some depth information compared to projective radiography on the other hand, digital tomosynthesis (DTS) was recently proved to be promising for guiding bronchoscopy interventions.^2^, ^3^, ^4^ However, DTS image quality is still limited for image guidance. DTS images are degraded by geometric distortions and streaking artifacts due to the limited angle acquisition (less than 50°) and the low number of projection images acquired (less than 75).

Patient‐specific computed tomography (CT) scans are typically performed prior to bronchoscopy procedures for diagnosis and/or planning the intervention. These priors are of high quality and provide a wealth of anatomical structures not apparent in intraoperative DTS images. The differences observed between preoperative scans and intraoperative scans are primarily attributed to patient and respiratory motions, as well as to the positioning of surgical tools. Consequently, when integrated effectively, prior CT scans can potentially augment intraoperative DTS image reconstruction with prior information. We have recently proposed a 3D/3D deformable registration algorithm customized for aligning prior CT scans to intraoperative DTS scans in bronchoscopy interventions.^5^ The proposed algorithm has been proved to be effective for correcting CT‐to‐body divergence.

Numerous iterative prior‐based image reconstruction (PBIR) methods have been proposed in the literature to address CT problems with insufficient data. A significant contribution in this domain is the work by Chen et al.,^6^ who introduced the prior image constrained compressed sensing (PICCS) algorithm. The main idea is to minimize an objective function seeking a sparse reconstructed image as well as a sparse difference between the reconstructed image and a previously acquired prior image (L1 norm minimization). This algorithm has been applied specifically to sparse‐view CT image reconstruction and has been limited to phantoms and static objects with DTS data.^7^ Similarly, Stayman et al.^8^ developed the prior‐image‐registered penalized‐likelihood estimator (PIRPLE), which integrates prior images into a model‐based penalized likelihood reconstruction of sparse‐view projection datasets. Notably, since some changes between the prior and current image can arise from motion between scans, a rigid registration step of the prior to the current anatomy was included in the reconstruction. Dang et al.^9^ extended this approach to a deformable PIRPLE (dPIRPLE), incorporating a 3D elastic deformation model into the model‐based iterative reconstruction method. In the context of sequential imaging scenarios, such as measuring tumor growth or visualizing surgical changes, Lee et al.^10^ proposed the region of change reconstruction strategy. This method computes the difference between the forward projections of a prior CT or CBCT image and the measured projections after registering the prior image to the current anatomy, emphasizing surgical changes. Pourmorteza et al.^11^ introduced the reconstruction of difference, which includes the prior image in the data fidelity term of the penalized‐likelihood cost function, unlike conventional PBIR techniques. For 4D intervention guidance, Kuntz et al.^12^ developed the prior image dynamic interventional computed tomography algorithm, allowing continuous reconstruction of time frame images from sparse‐view datasets and a high‐quality prior image. It addresses patient motion‐induced mismatches between scans and utilizes analytic reconstruction iteratively while imposing a constraint that restricts the desired information to only a small number of voxels with high absolute values. Flach et al.^13^ proposed the running prior technique, involving the registration of the prior image to the current anatomy and replacement of outdated projections with newly‐acquired ones. This work has been extended to include a deformable 2D/3D registration.^14^

Most of the aforementioned PBIR algorithms have been applied to sparse‐view CT or CBCT data rather than limited‐angle or DTS data. Additionally, while priors have typically been integrated into the body of the iteration of the reconstruction algorithm, the initialization has often been overlooked.

The poor depth resolution inherent to DTS is mainly due to a low undersampling in the Fourier domain.^15^ Only a small portion of the Fourier's space is sampled when projection data are acquired over a limited angular range. For instance, with a linear DTS motion, only a portion of Fourier's space formed of planes in a double‐wedge domain is sampled. The large unsampled region in the frequency domain leads to an ill‐conditioned reconstruction problem characterized by a vast nullspace, where a multitude of potential solutions could satisfy the same measured projection data. The algebraic reconstruction technique (ART) is an iterative method used to reconstruct images from incomplete data.^16^, ^17^ During the ART process, an initial estimation of the solution is established, followed by a series of iterative steps. These steps seek to minimize the difference between the measured data and the projections of the current image estimate. In the case of DTS image reconstruction, the yielded solution is heavily dependent on the initial guess.

Various options for initializing the iterative process have been proposed in the literature. These range from uniformly distributing zero values or very small positive values, to using a uniform distribution of averaged attenuation coefficients.^18^ Some methods suggest utilizing reconstruction results from other techniques like the backprojection method.^19^ In a particular study, an optimal initial image leveraging the symmetry of object contours was employed.^20^ However, it is worth noting that the assumption of object contour symmetry does not hold true in many medical CT images. Additionally, the symmetry axis of the object often does not align with the scanner axis due to positioning inaccuracies.

With a patient‐specific prior CT image available and a reliable deformable registration algorithm capable of aligning this prior CT image with the intraoperative DTS image, we have a valuable opportunity to utilize this properly‐registered prior CT image as an initial estimate for the iterative ART reconstruction. This particular form of initialization of DTS image reconstruction in the context of bronchoscopy interventions has not been previously published in the literature and serves as the focal point of this work. The subsequent sections provide a comprehensive presentation of the proposed prior‐aided DTS reconstruction algorithm, along with the experiments carried out on both a physical phantom and patient bronchoscopy data, as well as the outcomes observed.

## 2 MATERIALS AND METHODS

### 2.1 Prior‐aided DTS reconstruction technique

**FIGURE 1:**
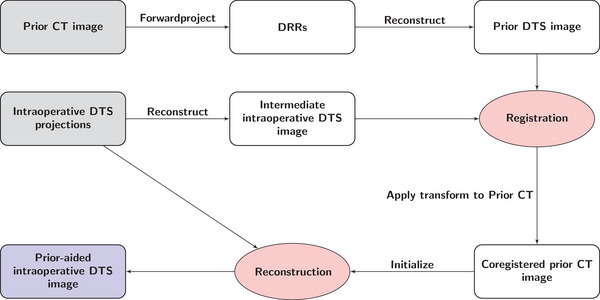
Flowchart of the proposed prior‐aided DTS reconstruction method. DTS, digital tomosynthesis.

Figure 1 illustrates the prior‐aided DTS reconstruction technique. The inputs are a prior CT image and a set of intraoperative DTS projection images. This set of projection images is first iteratively reconstructed yielding an intermediate intraoperative DTS image. ART is used with an ordered‐subsets scheme.^21^ At this stage, all the image voxels are initialized at zero. Due to the complex anisotropic resolution characteristics of DTS and the poor match in image content of CT and DTS images, a DTS‐to‐DTS registration is preferred over CT‐to‐DTS registration.^5^ Therefore, the prior CT image is forwardprojected using the same system geometry as the DTS yielding the digitally reconstructed radiographs (DRRs). A prior DTS image is obtained by reconstructing these DRRs using ART. This reconstructed image is registered to the intermediate intraoperative DTS image and then the registration transform is applied to the prior CT image to get a co‐registered prior CT image. Finally, the intraoperative DTS projections are iteratively re‐reconstructed using ART with the registered prior CT image set as a first guess.

### 2.2 Deformable CT‐to‐DTS image registration

In this work, we employed our recently proposed deformable registration algorithm aligning prior CT images to intraoperative DTS images in image‐guided bronchoscopy interventions.^5^ This algorithm is based on a multistage, multiresolution approach. Due to the complex dynamics in the chest region, a combination of affine and elastic B‐spline transformation models are employed at four different stages with bone and lung mask images. A multiresolution strategy with a Gaussian image pyramid is applied to the image data. Besides the image pyramid schedule, a multigrid strategy starting with a coarse control point grid at the first resolution level and gradually refined at the subsequent levels is embedded within the B‐spline transformation model. A cost function including the normalized correlation coefficient (NCC) is used for the affine transformation model, while a multimetric weighted cost function combining the NCC and the sum of squared tissue volume differences^22^, ^23^ is used for the B‐spline transformation model. For a detailed explanation of this registration algorithm, interested readers are referred to Saad et al.^5^

### 2.3 Data

#### 2.3.1 Physical phantom

**FIGURE 2:**
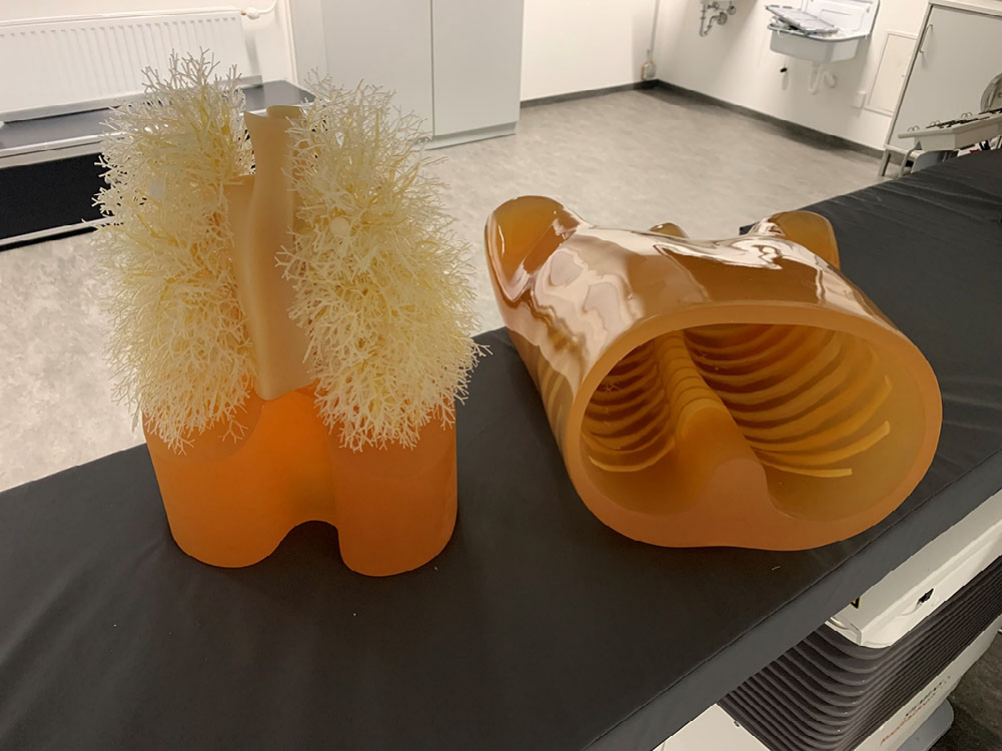
Anthropomorphic Lungman thoracic phantom from Kyoto Kagaku, Japan.

As a proof‐of‐concept, the Lungman chest phantom from Kyoto Kagaku Co., Ltd, Japan, served as the experimental model (Figure 2) in this study. This sophisticated chest phantom replicates a life‐sized human torso with intricate anatomical detail, including pulmonary arteries and airways. Although lacking lung parenchyma, it is filled with air and accurately mimics human chest anatomy. Various arrangements of synthetic spherical nodules were placed within its vascular model.

This phantom was first scanned with the SOMATOM X.cite scanner (Siemens Healthcare GmbH, Erlangen, Germany) at 80 KVp to obtain a prior CT volume. The resulting image had dimensions of 768 × 768 × 473 voxels, with a voxel size of 0.433 mm× 0.433 mm × 0.7 mm. To mimic an intraoperative scenario, a guide wire with a stent was inserted into the phantom, then it was placed at an arbitrary location on the angiography patient table and scanned using the ARTIS icono angiography system (Siemens Healthcare GmbH, Erlangen, Germany) at 90 KVp to acquire an intraoperative CBCT scan. The reconstructed CBCT image comprised 512×512×368 voxels, with a voxel size of 0.49 mm ×0.49 mm × 0.49 mm. Since this phantom is static and does not allow deformable motion, only the first two stages of the deformable registration algorithm^5^ are required in the registration step replacing the affine transform with a simple rigid transform.

**FIGURE 3:**
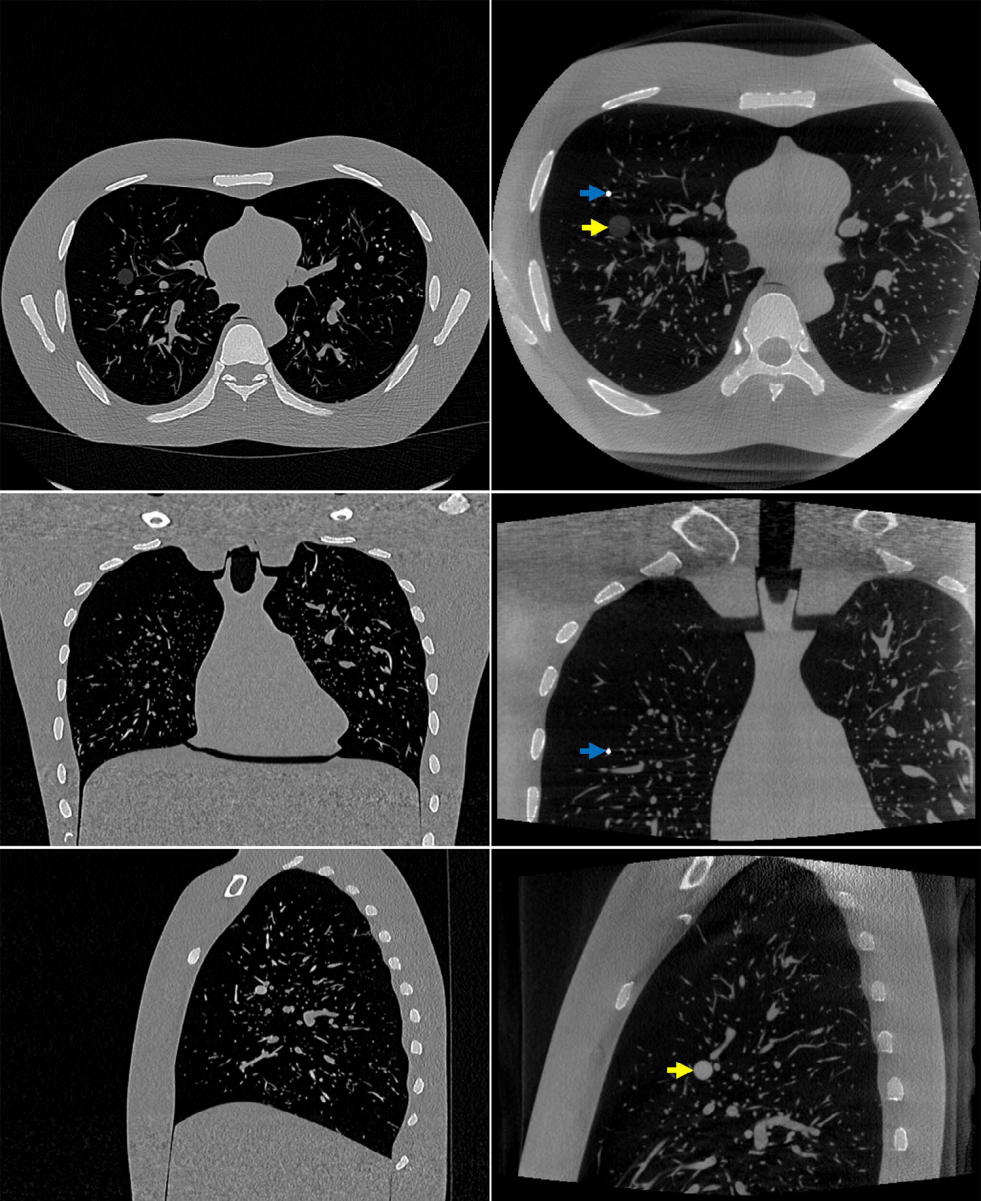
Lungman phantom data. (a) Prior CT and (b) intraoperative CBCT. Upper row: one axial slice. Middle row: one coronal slice. Lower row: one sagittal slice. The display window range is [−1000 HU, 1000 HU]. CBCT, cone‐beam computed tomography; CT, computed tomography.

Figure 3 illustrates the prior CT (a) and intraoperative CBCT (b) images of the Lungman phantom. Axial (upper row), coronal (middle row), and sagittal (lower row) slices are shown for each image. Slices of the intraoperative CBCT where a lesion (yellow arrows) or the guide wire (blue arrows) exists and the corresponding slices in the CT image are shown.

#### 2.3.2 Patient data

**FIGURE 4:**
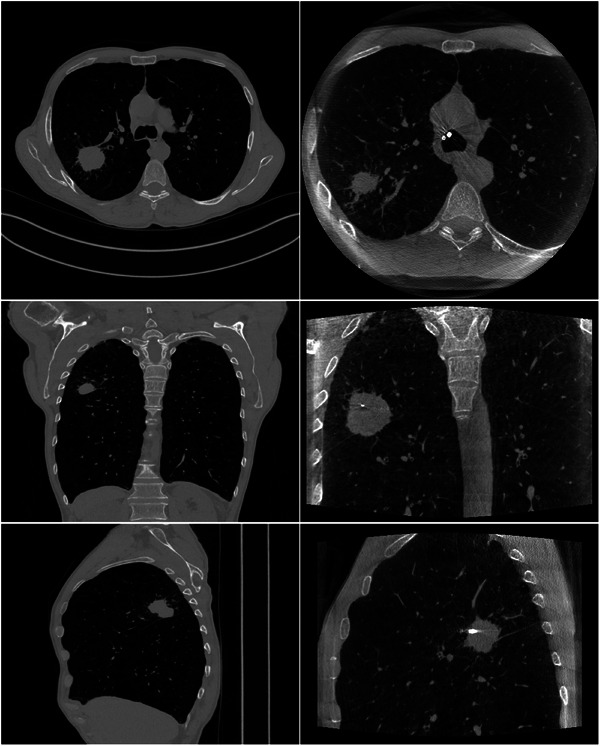
Patient bronchoscopy data. (a) Prior CT and (b) intraoperative CBCT. Upper row: one axial slice. Middle row: one coronal slice. Lower row: one sagittal slice. The display window range is [−1000 HU, 1000 HU]. CBCT, cone‐beam computed tomography; CT, computed tomography.

To take the diversity and complexity present in clinical scenarios into account, the proposed algorithm was tested on six patient bronchoscopy datasets. Each dataset comprises a preoperative CT volume and some intraoperative CBCT scans (projection images and reconstructed images). These data were obtained during CBCT‐guided bronchoscopy procedures. The planning CT images were obtained 4 days to 7 weeks prior to the actual procedures using devices from various manufacturers. During the procedure, intraoperative CBCT images were captured using the same C‐arm device (AXIOM Artis dTA, Siemens Healthcare GmbH, Erlangen, Germany). Typically, two to three CBCT scans were conducted at different stages of the procedure. For the purpose of this study, only the CBCT scans taken at the very end of the procedure to confirm tool‐in‐lesion were utilized. These CBCT images depict a flexible bronchoscope navigating through the patient's trachea towards the target lesion, with a transbronchial biopsy needle inserted into the lesion. It is worth noting that we have used these datasets in another study where patient demographics and acquisition parameters for each dataset are listed.^5^ Figure 4 portrays the original prior CT (a) and intraoperative CBCT (b) image of one of the patients. Axial (upper row), coronal (middle row), and sagittal (lower row) slices showing the target lesion and the interventional tool are shown for the intraoperative CBCT image. The corresponding slices are shown for the prior CT image. The main body of the paper focuses on the reconstructed images of this case, while the reconstruction results from other cases will be included in the quantitative analysis.

### 2.4 DTS data generation

**FIGURE 5:**
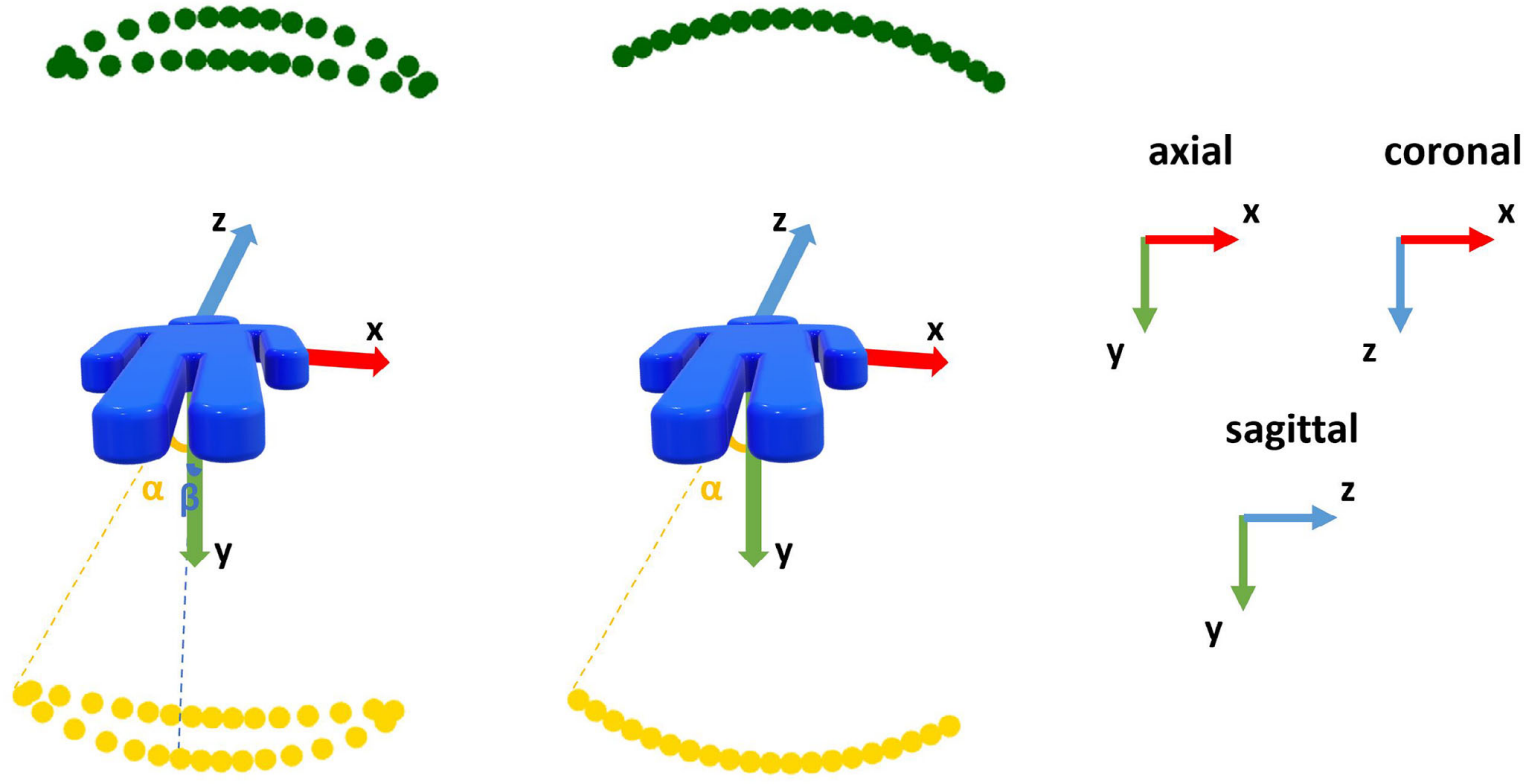
Illustration of the DTS scan orbits: (a) SE and (b) PL. The source trajectory is shown in yellow below the patient and the detector trajectory is shown in green above the patient. DTS, digital tomosynthesis; PL, pseudo‐linear; SE, spherical ellipse.

Since DTS data were not available at the time of writing, DTS projection images were generated from the CBCT data (volumes and projections) according to two scan geometries (Figure 5):

1. Spherical ellipse (SE) DTS scan geometry: DTS projection images were simulated by forwardprojecting the intraoperative CBCT images assuming a SE scan orbit. This scan geometry is customized for guiding bronchoscopy interventions.^4^ This simulation setup assumes the x‐ray source and the flat‐panel detector are mounted on a C‐arm system and each move along a SE trajectory. The x‐ray source moves beneath the patient table while the detector is positioned above the patient. The detector undergoes an in‐plane rotation ensuring its rows remain tangent to the scanning orbit. This orbit is characterized by two tomographic angles, a large angle α and a small angle β, with the latter oriented in the cranial‐caudal direction to avoid collision with robotic bronchoscope holders commonly employed in interventional bronchoscopy procedures. The SE acquisition geometry is implemented in the CTL,^24^ an open‐source toolkit. The source‐to‐isocenter distance and the source‐to‐detector distance are fixed at 785 mm and 1200 mm, respectively. The simulation assumes a flat‐panel detector with dimensions of 616×480 pixels and a pixel pitch of 0.616 mm. To mimic real‐world conditions, Poisson noise is incorporated into the projection data, with a photon flux set at $4.75\times 10^{8}$ photons per cm^2^. Additionally, limited detector dynamics are simulated, introducing a saturation effect to cap all extinction values at 20.
2. Pseudo‐linear (PL) DTS scan geometry: A limited set of projection images was selected from the original projection images of the CBCT acquisitions. The selected projection images cover a limited angular range of ±α. In order to reconstruct the DTS images, the projection data underwent the same preprocessing procedure utilized for the original projection images at the AXIOM Artis dTA workstation. This procedure encompasses converting raw intensity data into extinction values ($\ln\frac{\mathrm{Initial}\;\mathrm{Intensity}}{\mathrm{Intensity}}$) and implementing various correction steps, primarily aimed at mitigating scatter and beam‐hardening distortions.

In this work, α and β were set to 23^0^ and 15^0^, respectively, and the number of projection views N was set to 72. These settings were shown to be optimal in our previous works.^4^, ^5^

### 2.5 Evaluation metrics

To evaluate the prior‐aided DTS reconstruction technique, reconstructed images are evaluated qualitatively by visual inspection and quantitatively by computing Pearson's correlation (PC). PC of two variables x and y is given by

(1) $$\mathrm{PC}=\frac{1}{n-1}\sum_{i=1}^{n}\left(\frac{x_{i}-\bar{x}}{s_{x}}\right)\left(\frac{y_{i}-\bar{y}}{s_{y}}\right)$$

where n is the sample size, $\bar{x}$ and $s_{x}$ are the mean and standard deviation of x, respectively, and $\bar{y}$ and $s_{y}$ are the mean and standard deviation of y, respectively. We used PC since it is not affected by differences in contrast between DTS and CBCT images.^25^ The proposed algorithm is compared to a standard zero‐initialized ART reconstruction. Both reconstructions were stopped after 50 iterations.

## 3 RESULTS

**FIGURE 6:**
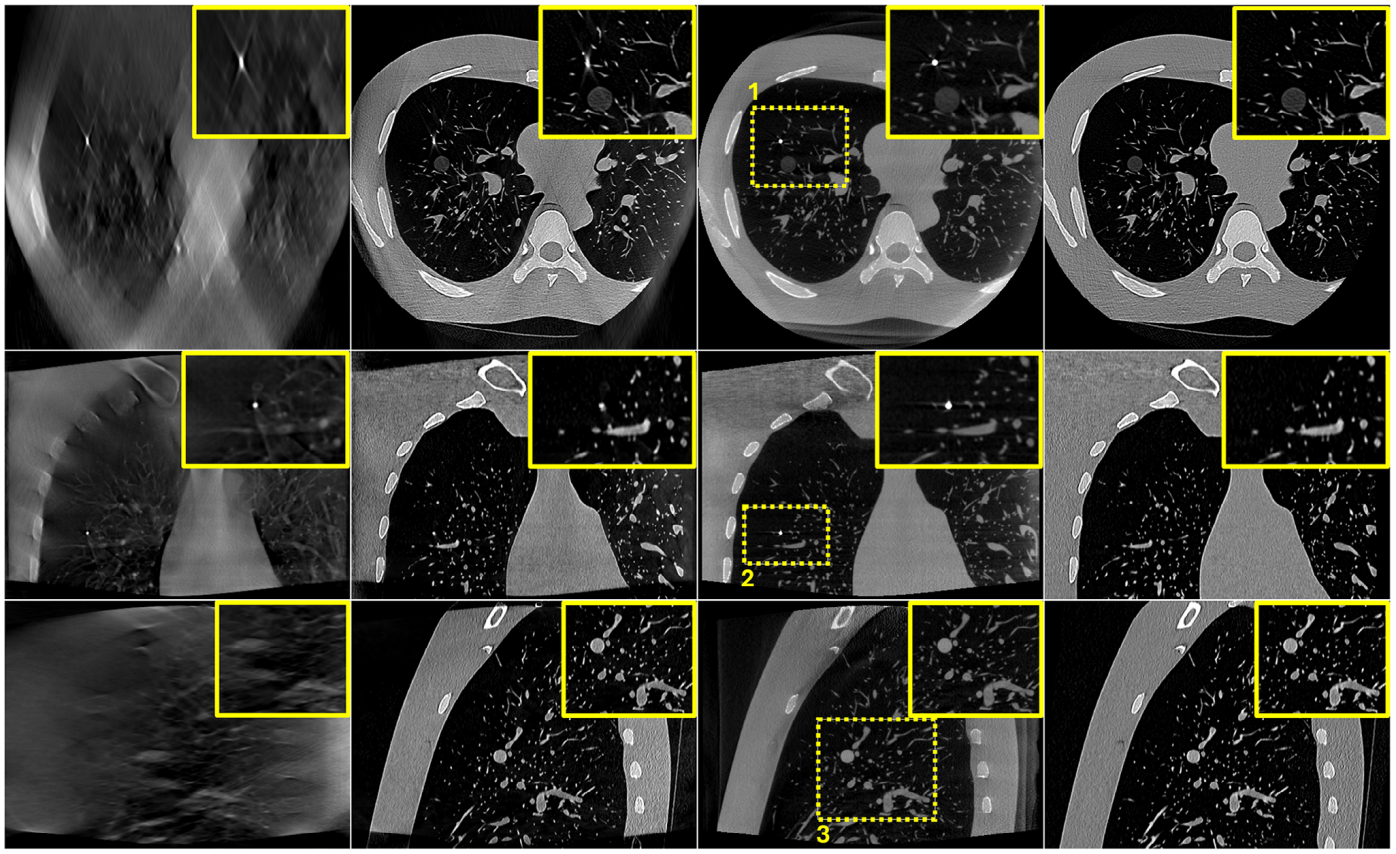
Lungman phantom reconstruction results using SE DTS data. (a) zero‐initialized ART, (b) prior‐aided reconstruction, (c) intraoperative CBCT, and (d) registered prior CT. Upper row: one axial slice. Middle row: one coronal slice. Lower row: one sagittal slice. Same slices are shown in (a), (b), (c), and (d). The display window range is [−1000 HU, 1000 HU]. ART, algebraic reconstruction technique; CBCT, cone‐beam computed tomography; CT, computed tomography; DTS, digital tomosynthesis; SE, spherical ellipse.

Figure 6 portrays the reconstructed images of the Lungman phantom using simulated DTS data according to a SE scan geometry as described in Section 2.4. The intraoperative DTS image reconstructed using a simple zero‐initialized ART (a) and the prior‐aided DTS reconstruction technique (b) are illustrated. For comparison, the intraoperative CBCT image (c) is shown as a reference and the co‐registered prior CT image (d) as well. Regions of interest (ROI) highlighted in yellow and surrounding critical structures are zoomed‐in and shown in the corner of each image. The zero‐initialized ART reconstruction exhibits poor depth resolution. This is mainly visible in the axial and sagittal slices. The target nodule is blurred and barely visible. It is difficult to correctly localize it in the depth direction. However, the prior‐aided reconstruction yields an image with an improved visibility. The target nodule can be distinguished from the lung background, and the resemblance between the CBCT image and the prior‐aided DTS image is considerably improved.

**FIGURE 7:**
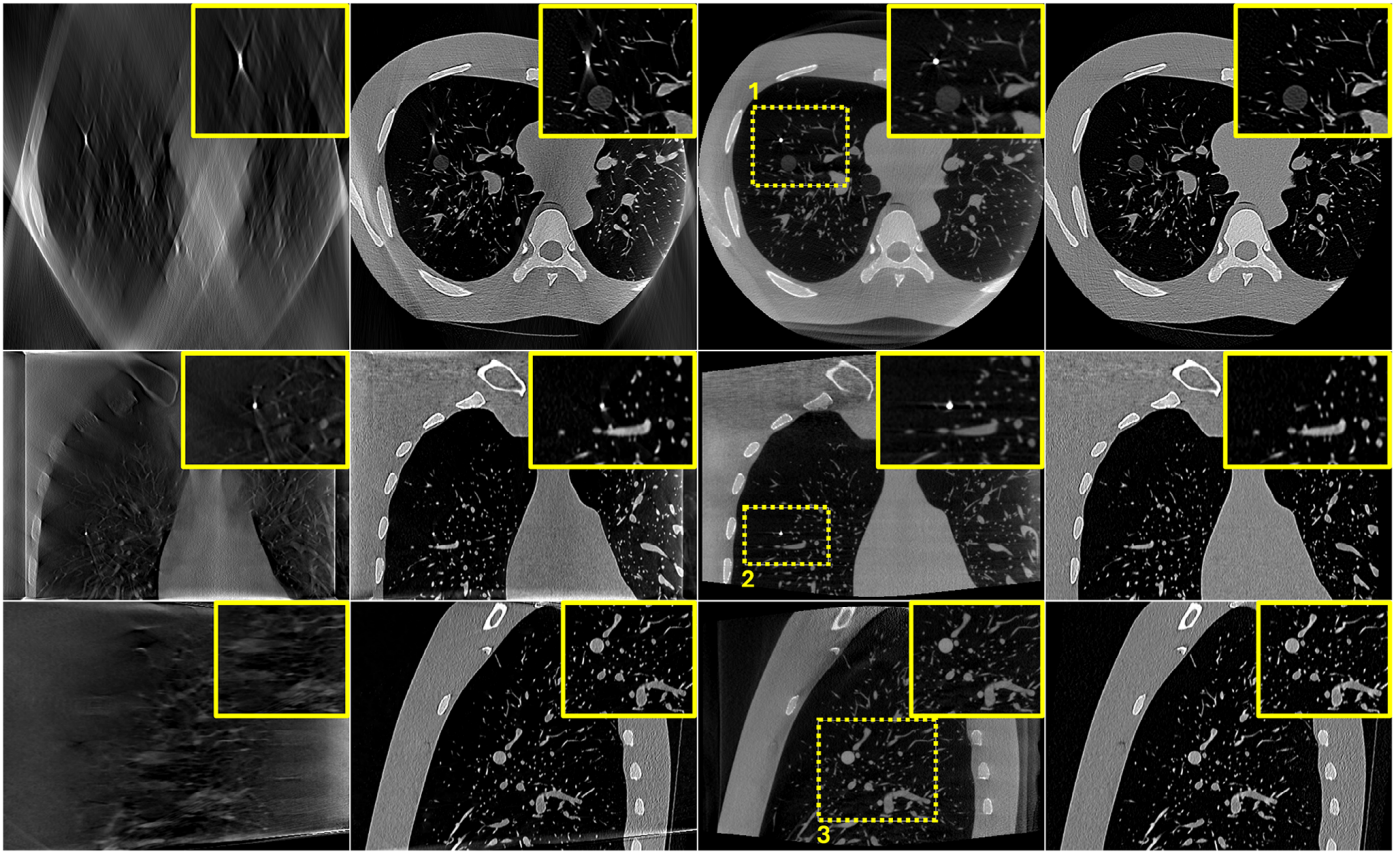
Lungman phantom reconstruction results using PL DTS data. (a) zero‐initialized ART, (b) prior‐aided reconstruction, (c) intraoperative CBCT, and (d) registered prior CT. Upper row: one axial slice. Middle row: one coronal slice. Lower row: one sagittal slice. Same slices are shown in (a), (b), (c), and (d). The display window range is [−1000 HU, 1000 HU]. ART, algebraic reconstruction technique; CBCT, cone‐beam computed tomography; CT, computed tomography; DTS, digital tomosynthesis; PL, pseudo‐linear.

In order to assess the proposed technique in real clinical conditions where many physical factors affect image quality, the proposed algorithm was applied on real DTS data of a PL scan geometry. The reconstructed images are portrayed in Figure 7. Same as in the case of SE data, a considerable enhancement in image quality is noticeable when using the prior‐aided reconstructions. No significant difference is noticed between simulated SE and real PL data.

To quantitatively assess these observations, PC between each reconstructed DTS image and the CBCT image was computed in the different selected ROI. These results are reported in Table 1. In accordance with the visual inspection, prior‐aided reconstructions exhibit considerably higher PC coefficients in all three ROIs compared to zero‐initialized reconstructions. No significant difference is observed between SE and PL.

**TABLE 1:** Lungman phantom quantitative results. PC computed in the different ROIs highlighted in yellow in Figures and using SE and PL data.

		SE	PL
ROI#1	Zero‐initialized	0.563	0.501
	Prior‐aided	**0.658**	**0.643**
ROI#2	Zero‐initialized	0.598	0.568
	Prior‐aided	**0.724**	**0.729**
ROI#3	Zero‐initialized	0.563	0.507
	Prior‐aided	**0.881**	**0.857**

*Note*: PC computed in the different ROIs highlighted in yellow in Figures 6 and 7 using SE and PL data. Highest PC values are indicated in bold.

Abbreviations: PC, Pearson correlation; PL, pseudo‐linear; SE, spherical ellipse.

**FIGURE 8:**
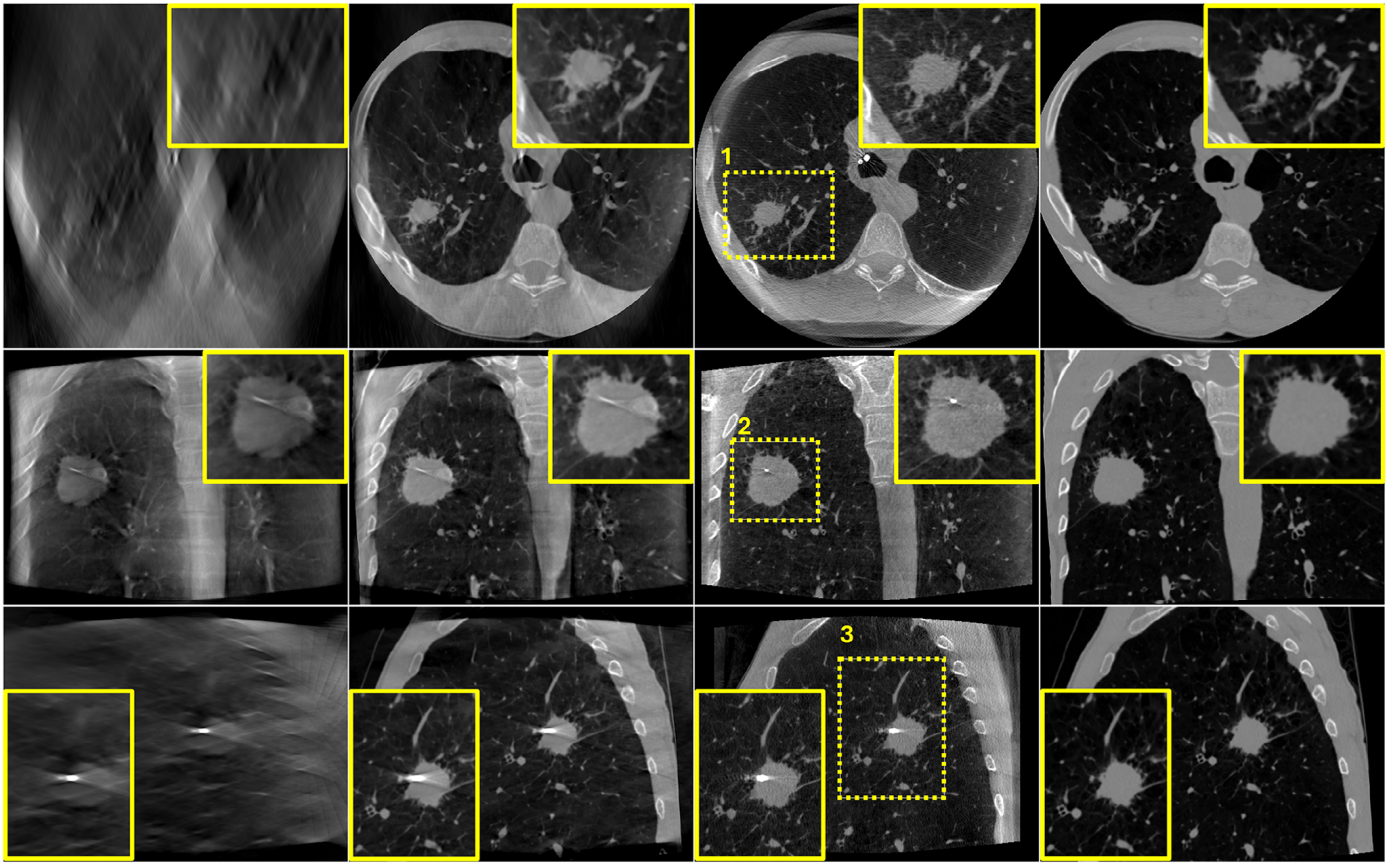
Patient bronchoscopy data reconstruction results using SE DTS data. (a) zero‐initialized ART, (b) prior‐aided reconstruction, (c) intraoperative CBCT, and (d) registered prior CT. Upper row: one axial slice. Middle row: one coronal slice. Lower row: one sagittal slice. Same slices are shown in (a), (b), (c), and (d). The display window range is [−1000 HU, 1000 HU]. ART, algebraic reconstruction technique; CBCT, cone‐beam computed tomography; CT, computed tomography; DTS, digital tomosynthesis; SE, spherical ellipse.

**FIGURE 9:**
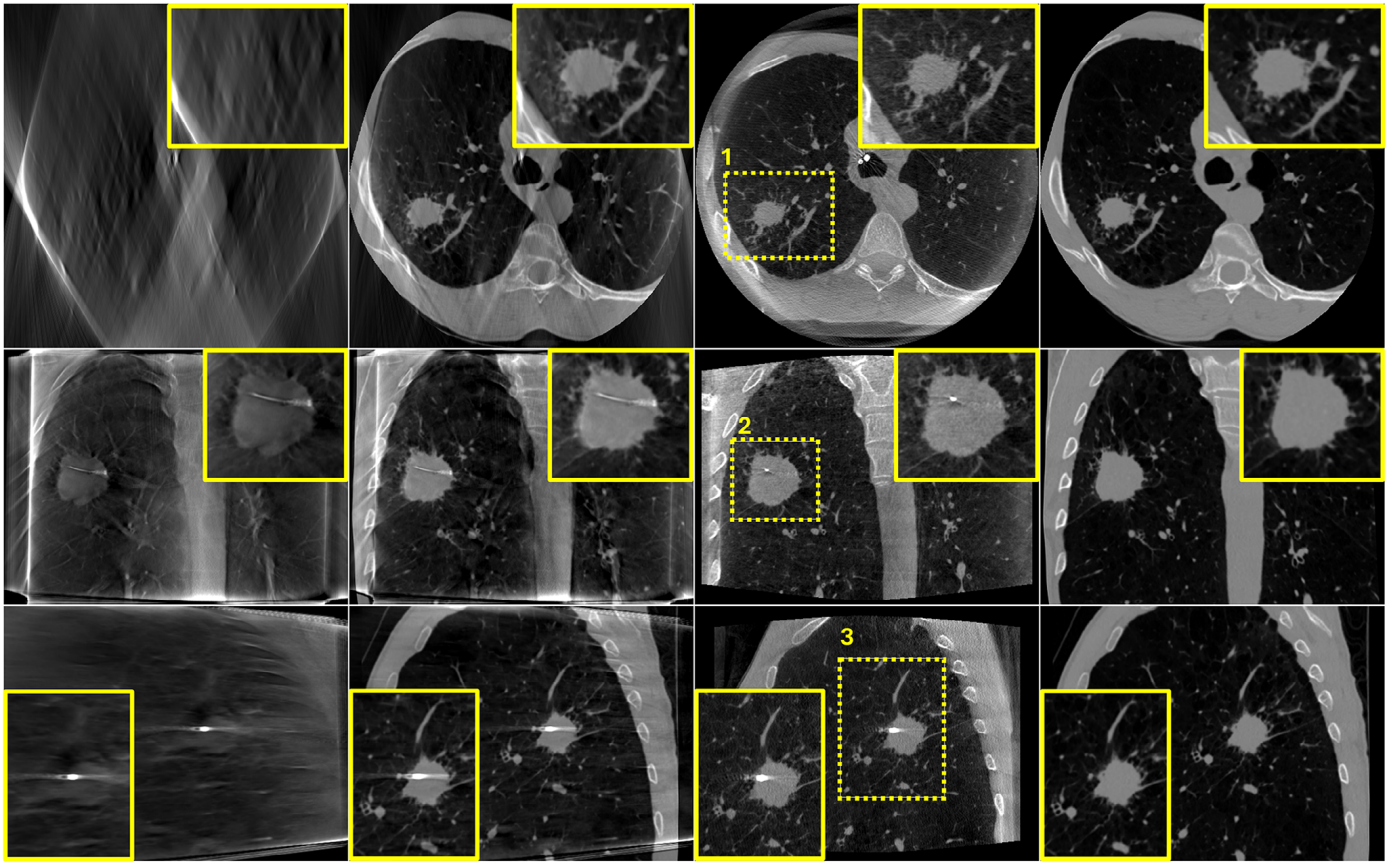
Patient bronchoscopy data reconstruction results using PL DTS data. (a) zero‐initialized ART, (b) prior‐aided reconstruction, (c) intraoperative CBCT, and (d) registered prior CT. Upper row: one axial slice. Middle row: one coronal slice. Lower row: one sagittal slice. Same slices are shown in (a), (b), (c), and (d). The display window range is [−1000 HU, 1000 HU]. ART, algebraic reconstruction technique; CBCT, cone‐beam computed tomography; CT, computed tomography; DTS, digital tomosynthesis; PL, pseudo‐linear.

To assess the performance of the algorithm in real clinical conditions where more complex data are encountered and where dynamic changes occur between prior CT scans and intraoperative DTS scans, the proposed algorithm was evaluated on the six bronchoscopy datasets described in Section 2.3.2. Figures 8 and 9 illustrate the reconstruction results of one of the patient using DTS data generated according to an SE and a PL scan geometry, respectively. A zero‐initialized reconstruction (a), a prior‐aided reconstruction (b), the reference CBCT (c), and the co‐registered prior CT (d) are shown in each figure. Same as in the previous experiment, ROIs surrounding the target lesion are highlighted and showed enlarged in the corner of each image. In accordance with the phantom‐based experiment, prior‐aided reconstructions provide images with a significantly improved quality compared to zero‐initialized reconstructions. The target lesion is blurred and barely discernible in the zero‐initialized reconstructions. The intervertebral disks and the pulmonary vasculature are obscured by the overlapping ribs. In Figure 8, prior‐aided reconstructions successfully recover these structures and exhibit an improved removal of out‐of‐plane artifacts. It is worth to be noted that the prior‐aided reconstruction using real PL data (Figure 9) shows slight differences to the intraoperative CBCT in the axial slice. This is mainly observable in the vertebrae and heart's region. During bronchoscopy interventions, these regions are not essential for accurate guidance. The lesion's region holds much greater importance for navigation, and as such, any reconstruction error outside it does not impact the intervention guidance. A slight difference is observed in the zoomed‐in lesion's region as well. This difference is also inherited from a slight misregistration error. These differences are not observed with the simulated SE data (Figure 8) as we have demonstrated in our previous work that the registration is slightly less accurate with real PL data.^5^

In Table 2, the PC coefficients between the reconstructed DTS image and the reference CBCT image for each of the six patient datasets are listed. PC coefficients were computed in three ROIs drawn in axial, coronal, and sagittal slices surrounding the target lesion. Consistent with the visual inspection, prior‐aided reconstructions exhibit remarkably higher PC values compared to zero‐initialized reconstructions in all three slices and for all six patients. This is valid for simulated SE data and real PL data. PC coefficients are just slightly higher with SE compared to PL for a few cases. This is expected as we have demonstrated in Ref. [5] that our registration algorithm exhibits a marginally lower level of accuracy when using real data as compared to simulated data. The variations in PC values across the six datasets are likely influenced by the specific anatomical structures included in the ROI where PC was computed. In some cases, the lesions were situated very close to bony structures, such as the ribs, which were included in the ROI. In contrast, in other cases where the lesion was located farther from the ribs or other bony structures, these high‐contrast structures were not included in the ROI, leading to differences in the PC values. Moreover, the position and angle of the bronchoscope during the procedure differ between patients, and these highly affect the appearance of the bronchoscope and the biopsy needle in the reconstructed DTS images leading to patient‐specific differences in the PC coefficients.

**TABLE 2:** Patient bronchoscopy data quantitative results.

Case	SE							PL					
	Zero‐initialized			Prior‐aided				Zero‐initialized			prior‐aided		
	Axial	Coronal	Sagittal	Axial	Coronal	Sagittal		Axial	Coronal	Sagittal	Axial	Coronal	Sagittal
1	0.497	0.691	0.436	**0.754**	**0.746**	**0.557**		0.443	0.592	0.417	**0.748**	**0.652**	**0.559**
2	0.747	0.677	0.690	**0.865**	**0.741**	**0.779**		0.621	0.561	0.548	**0.692**	**0.576**	**0.678**
3	0.638	0.347	0.640	**0.842**	**0.908**	**0.860**		0.597	0.204	0.488	**0.795**	**0.701**	**0.745**
4	0.566	0.807	0.621	**0.821**	**0.881**	**0.738**		0.494	0.736	0.484	**0.679**	**0.752**	**0.669**
5	0.487	0.778	0.733	**0.905**	**0.908**	**0.806**		0.521	0.719	0.649	**0.761**	**0.861**	**0.685**
6	0.707	0.813	0.681	**0.903**	**0.933**	**0.800**		0.606	0.689	0.605	**0.844**	**0.867**	**0.719**
Average	0.607	0.686	0.634	**0.848**	**0.853**	**0.757**		0.547	0.584	0.532	**0.753**	**0.735**	**0.676**

*Note*: PC between the reconstructed DTS image and the reference CBCT image computed in the different ROI surrounding the target lesion for the six datasets. Results with simulated SE DTS data and real PL DTS data are listed. Highest PC values are indicated in bold.

Abbreviations: PC, Pearson correlation; PL, pseudo‐linear; SE, spherical ellipse.

## 4 DISCUSSION

In this work, we investigated the use of a prior‐aided DTS reconstruction technique to enhance intraoperative DTS images for image‐guided bronchoscopy procedures. Building upon a recently published deformable CT‐to‐DTS registration algorithm,^5^ our focus was primarily on optimizing the initialization of the iterative ART reconstruction algorithm with a well‐aligned prior CT image. This patient‐specific image is often of higher resolution and shares a fair amount of anatomical information with the intraoperative DTS image. However, this information is poorly visualized in the DTS image due to the limited‐angle acquisition and subsequently the strong limited‐angle artifacts. Prior CT images are a promising source to compensate for this missing data. The prior‐aided DTS reconstruction technique depends upon using the registered prior CT image as a first guess of the iterative ART reconstruction. Experiments conducted on a physical phantom and patient bronchoscopy data and outcomes evaluated through qualitative and quantitative analyses showcased a substantial advancement with the prior‐aided DTS reconstruction over the standard zero‐initialized ART reconstruction. This holds true for DTS data simulated according to a SE scan geometry and real DTS data generated according to a PL scan geometry as well. Our proposed technique notably enhanced depth resolution and visibility of the different chest structures, mainly of the target lesion and its vicinity, bringing the reconstructed DTS images remarkably close to intraoperative CBCT images. These findings were corroborated not only through visual inspection but also through quantitative assessment, including the computation of PC coefficients.

Although the initial estimation in iterative algorithms is often overlooked in its significance, we underscored its critical importance, particularly in addressing such inherently underdetermined systems, like in DTS reconstruction. Through our exploration, we demonstrated the profound advantages of integrating prior knowledge into the DTS reconstruction process. This integration yielded a remarkable improvement in the visibility of various anatomical structures, and successfully transformed DTS images from a radiography‐like quality to a more tomography‐like appearance, bringing them to a level comparable to CBCT images. Notably, this enhancement was achieved using just 72 projection images acquired with a maximal tomographic angle of 23°, alongside a prior CT image. This corresponds to a radiation dose reduction of almost 81.8% compared to a CBCT acquisition since the latter requires around 397 projection images.

It is worth reiterating that our approach differs from prior‐based methods found in the literature. Instead of incorporating the prior throughout the reconstruction process, we utilize it solely during the initialization, which is often an overlooked step. By employing a straightforward ART reconstruction method rather than integrating the prior as a regularization term throughout the iteration, our algorithm is expected to significantly reduce computational complexity. In its current implementation, the registration step using our proposed algorithm^5^ takes approximately 10 minutes, and the reconstruction of one DTS volume takes approximately 75 seconds. These computations are conducted on a 64‐bit desktop computer featuring an Intel(R) Core(TM) i7 CPU @ 3.60 GHz and 32 GB RAM. Transitioning the computation of the registration to GPUs and employing optimizers with accelerated convergence properties, coupled with advancements in computing power, promises to curtail computation time substantially, facilitating real‐time CT‐augmented DTS during interventions. In future work, PBIR techniques, such as PICCS,^7^ will be modified and adapted to suit our complex clinical bronchoscopy data, and a comparison to our proposed prior‐aided reconstruction technique will be conducted.

An area of concern regarding the proposed prior‐aided DTS reconstruction algorithm is its sensitivity to misregistration errors in challenging cases. Instances of such challenges include complications like atelectasis, significant truncation artifacts, and substantial movements or deformations of lesions between scans. By the design of the prior‐aided reconstruction technique, information obtained from the DTS acquisition are incorporated in the prior‐aided ART reconstruction, while any missing information is inherited from the co‐registered prior CT image. In cases where registration inaccuracies occur, there is a potential for misleading conclusions during image interpretation. To address this potential risk, future work will involve clinical experts in the evaluation process. We have aimed to devise a bronchoscopy guidance protocol that exclusively relies on preoperative CT scans and intraoperative DTS scans, aiming to supplant the conventional use of intraoperative CBCT scans in bronchoscopy procedures. In cases where CT‐to‐DTS image registration may encounter challenges, an alternative guidance protocol based on starting the bronchoscopy procedure with a CBCT scan, followed by subsequent DTS scans is a potential alternative. The CBCT image can be used instead of the prior CT image. It can be registered to subsequent DTS images and then used as an initialization of the ART reconstruction to improve DTS image quality.

The accurate visualization of the position and shape of the biopsy needle is crucial for a proper guidance of bronchoscopy interventions. While the overall image quality is improved by our proposed reconstruction method, the appearance of the bronchoscopy tools does not show significant improvements. As these tools are not present in the prior CT image, they are reconstructed in the intraoperative DTS image just based on the DTS acquisition. To enhance their appearance, prior information about these interventional tools (e.g., volume, shape, or material of the bronchoscope) could be exploited besides using preoperative CT images as prior knowledge.

In future studies, real DTS data of physical phantoms will be acquired on flexible DTS trajectories and will be compared to CBCT data. While we have shown that CT‐augmented DTS is a promising alternative to CBCT in guiding bronchoscopy procedures, it is certainly imperative to conduct clinical studies comparing the diagnostic yield of CBCT‐guided bronchoscopy to that of DTS‐guided bronchoscopy before fully integrating DTS as a primary image guidance modality in bronchoscopy procedures.

## 5 CONCLUSION

In this work, we proposed a prior‐aided DTS reconstruction technique to augment intraoperative DTS images with prior CT images for guiding bronchoscopy interventions. This technique incorporates a properly registered prior CT image in the initialization step of the iterative DTS reconstruction algorithm. Experiments on a physical phantom as well as on patient bronchoscopy data indicated a significant improvement in DTS image quality with the proposed reconstruction technique compared to the standard DTS reconstruction technique. This work lays the foundation for DTS‐guided bronchoscopy interventions.

## CONFLICT OF INTEREST STATEMENT

The authors declare no conflicts of interest.
